# Mapping the lipidomic secretome of the early equine embryo

**DOI:** 10.3389/fvets.2024.1439550

**Published:** 2024-10-04

**Authors:** Edwina F. Lawson, Russell Pickford, Robert John Aitken, Zamira Gibb, Christopher G. Grupen, Aleona Swegen

**Affiliations:** ^1^School of Environmental and Life Sciences, College of Engineering, Science and the Environment, University of Newcastle, Callaghan, NSW, Australia; ^2^Bioanalytical Mass Spectrometry Facility, University of New South Wales, Sydney, NSW, Australia; ^3^Sydney School of Veterinary Science, Faculty of Science, The University of Sydney, Camden, NSW, Australia

**Keywords:** lipidomics, lipids, embryo, equine, pregnancy, *in vitro*

## Abstract

The lipidomic secretions of embryos provide a unique opportunity to examine the cellular processes of the early conceptus. In this study we profiled lipids released by the early equine conceptus, using high-resolution mass spectrometry to detect individual lipid species. This study examined the lipidomic profile in embryo-conditioned media from *in vivo*-produced, 8–9 day-old equine embryos (*n* = 3) cultured *in vitro* for 36 h, analyzed over 3 timepoints. A total of 1,077 lipid IDs were recorded across all samples, containing predominantly glycerolipids. Seventy-nine of these were significantly altered in embryo conditioned-media versus media only control (*p* < 0.05, fold-change >2 or < 0.5). Fifty-five lipids were found to be released into the embryo-conditioned media, of which 54.5% were triacylglycerols and 23.6% were ceramides. The sterol lipid, cholesterol, was also identified and secreted in significant amounts as embryos developed. Further, 24 lipids were found to be depleted from the media during culture, of which 70.8% were diacylglycerols, 16.7% were triacylglycerols and 12.5% were ceramides. As lipid-free media contained consistently detectable lipid peaks, a further profile analysis of the various components of non-embryo-conditioned media consistently showed the presence of 137 lipids. Lipid peaks in non-embryo-conditioned media increased in response to incubation under mineral oil, and contained ceramides, diacylglycerols and triacylglycerols. These results emphasize the importance of a defined embryo culture medium and a need to identify the lipid requirements of the embryo precisely. This study sheds light on early embryo lipid metabolism and the transfer of lipids during *in vitro* culture.

## 1 Introduction

Early pregnancy is one of the most challenging and enigmatic facets of mammalian reproduction. It comprises a precarious pre-implantation phase, in which the embryo is yet to develop placental attachments and the mother is yet to establish stable systemic support of the pregnant state. The early conceptus relies on localized embryo-maternal interactions for survival, including signaling its presence to the maternal environment and in turn receiving metabolic support through endometrial secretions. In women, pre-implantation embryo loss is estimated at 10–40% and overall pregnancy loss from fertilization to birth is approximately 40–60% (1). The equine pregnancy poses similar challenges with an estimated 20–30% of conceptions failing prior to implantation (2, 3). The role of embryo-maternal communications is particularly pertinent in the horse, as this species features a long period (40–45 days) before the development of definitive placentation (4), while the signal that facilitates maternal recognition of pregnancy (MRP) remains undiscovered (5). Along with proteins and miRNA, lipids are anticipated to play a key role in both signaling between embryo and endometrium, as well as in nourishing and supporting the early embryo during this early period (6). While the abundance and function of some of these compounds in the embryo environment have begun to be investigated, the secreted lipids engaged in embryo-maternal signaling have yet to be profiled in any mammalian species.

For studying Assisted Reproductive Technology (ART), the horse is a fitting model for human clinical procedures. Women and mares are both mono-ovulatory, and have similar follicular dynamics and embryo developmental kinetics until the blastocyst stage (7). In addition, both species share infertility concerns due to obesity and aging (8–12), which are believed to influence the lipid composition of the oocyte. In comparison with many other species, the lipid content of equine oocytes and embryos is notably higher (13, 14). Embryonic cytoplasmic lipid droplets, in the form of triglycerides, represent the most abundant energy source and are accumulated during embryo development (15). In addition, the embryos of both equine and human species are particularly sensitive to both ambient and culture environments (16–18). In humans, it has been suggested that microtubule spindles are thermosensitive (19), and spindle integrity can be irreversibly altered by temperature (20). This sensitivity to the culture environment is particularly pertinent for the mare, where effective *in vitro* fertilization (IVF) has proven to be a major challenge and is not yet commercially available (21, 22). As such, intra-cytoplasmic sperm injection (ICSI) is currently the only commercial method for producing equine embryos *in vitro*. Reciprocal knowledge May be gained through the comparative study of equine and human ART and infertility treatments, helping to define the optimum *in vitro* requirements for oocyte maturation and embryo culture.

Lipids are expected to play roles in embryo maternal signaling. They regulate reproductive cyclicity and are intrinsically linked to pregnancy [reviewed in Lawson et al. (14)]. With the high metabolic rate of the early embryo, functionally, lipids serve as a primary energy source, but, they also serve as a molecular membrane scaffold that regulates cellular signaling (23). Acting primarily through their interactions with proteins, many of the pathways by which lipids modulate these proteins are not yet fully understood (24). Hence, understanding lipid biosynthesis and hormone structure will lay the groundwork for better understanding embryonic requirements and how this May influence maternal signaling. In equine embryos, researchers have previously identified proteins secreted by the early equine embryo (25–29), and attempted to identify a putative MRP factor secreted by the conceptus (30, 31). In a previous study of the protein component of the embryo secretome it was found that proteins involved in lipid-associated and lipoprotein function were consistently over-represented (29). Embryo-produced mediators, such as the phospholipid Platelet-activating factor (PAF) have been suggested to have an early stimulatory effect (32, 33) and precursors such as arachidonic acid and docosahexaenoic acid, which are essential constituents of membrane lipids in other species, have been postulated to play a crucial role in equine embryonic development (34). Such findings suggest lipids are potential regulators of the embryo-signaling response and, as such, warrant investigation into the lipidomic profile of equine embryos for investigations into MRP. This leads to the hypothesis that lipids themselves and the interactions between proteins and lipids have important roles in embryo-maternal signaling as well as equine embryo development.

Apart from maternal signaling, at the pre-implantation stage of development, embryos require the biosynthesis of lipids, particularly for energy metabolism, cell membrane construction and signaling events involved in gene activation (34). It is well established that until approximately day 22 after ovulation, the equine embryo is encased in a glycoprotein capsule (35), which covers the equine blastocyst after it loses its zona pellucida (36). The capsule is believed to play a protective role, and participate in fetal-maternal interface communication (37). Importantly for the study of lipids resent research suggests that exosomes and other extracellular vesicles secreted by the conceptus membranes May play important roles in equine embryo-maternal communication during this early period (38). However, despite this capsule embryos are able to produce and continue to secrete prostaglandin E2 and other prostaglandins, such as PGF2α and PGI_2_ (39–41). More recently, prostaglandin synthesis enzymes were shown to be involved in embryo-driven forward motion motility, due to their location on the “peri-embryonic” pole (42). Prostaglandins are lipid autacoids derived from arachidonic acid by the cleavage action of phospholipase A2s (PLA2s). PLA2s are known to be functionally involved in diverse cellular events, including phospholipid metabolism, immune functions and signal transduction, and their actions generate bioactive lipid mediators (43). Such *de novo* biosynthesis of lipids indicates that equine embryos autotrophically produce their own lipid supply, contributing directly to the steroid environment of the intrauterine lumen (44). Such findings implicate the role of lipids and protein–lipid complexes in supporting the early equine embryo, particularly at the pre-implantation stage. As the lipidomic component of embryonic secretions have not been well described, defining what lipids are released by embryos will help delineate the signaling pathways important for a successful pregnancy. Therefore, this research sought to comprehensively describe the profile of lipids released by early equine embryos using high-resolution mass spectrometry.

Lipidomics is a systems-level analysis and characterization of lipids. Like other omics technologies, such as transcriptomics or proteomics, lipidomics is a global profiling of lipid species present in cells, tissues or extracted body fluids (45). The applications of lipidomics technology are rapidly evolving and currently it allows detection of a broad range of lipid classes, categories, and quantification of lipid species. The approach has also provided valuable information on biomarkers for disease pathophysiology, and shed light on the detailed biophysiological functions involved in reproductive biology (46). Currently, the LIPID MAPS® classification system organizes lipids into eight categories: fatty acyls, glycerolipids, glycerophospholipids, sphingolipids, sterol lipids, prenol lipids, saccharolipids and polyketides. Each category can be divided into numerous classes with individual lipid identities (47). Recent advances in mass spectrometry-based lipidomics technology have meaningfully improved the detection of a vast array of lipids (48). The technology allows for the detection of minute, yet biologically significant fluctuations in lipid levels. For example, it has recently been used to profile the fatty acid content of spermatozoa from different species (49). As such, the precision of lipidomic technology now offers opportunities to answer many of the remaining unanswered questions. Therefore, in this study we aim to profile the lipids secreted by the early equine embryo in an *in vitro* setting, and in addition, to examine the embryo culture model, by carrying out lipidomic profiling of embryo culture media.

## 2 Materials and methods

### 2.1 Artificial insemination

All procedures undertaken in this study were reviewed by the University of Newcastle Animal Ethics Committee and approved under the Australian code for the care and use of animals for scientific purposes (approval number A-2018-804). Standardbred mares (*n* = 3) were housed on pasture. Mares’ reproductive cycles and ovulations were monitored by transrectal palpation and ultrasonography. Upon signs of impending ovulation, ovulation was induced with a synthetic analog of gonadotrophin-releasing hormone (GnRH), then inseminated with semen containing at least 5 × 10^8^ motile spermatozoa, obtained from one of three fertile stallions, extended in SpermSafe (University of Newcastle, Callaghan, Australia), and stored for up to 3 days at 17°C. All mares received the same routine post-mating treatment consisting of an intrauterine infusion with 1 L of saline followed 8 h later by an intramuscular administration of oxytocin.

### 2.2 Embryo collection and culture

**Figure 1:**
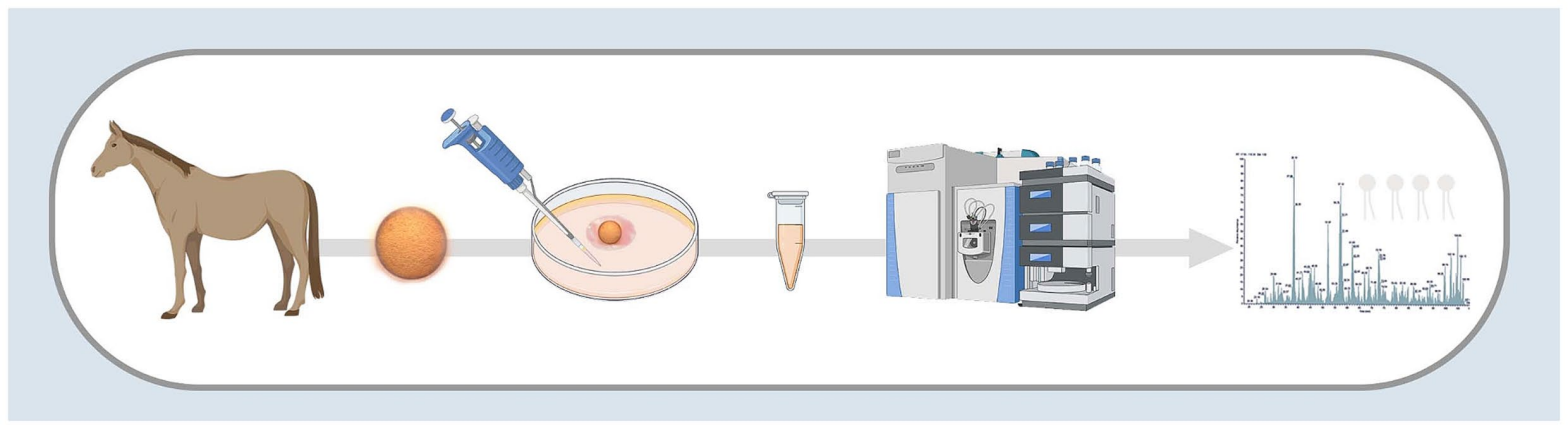
Visual summary of methods used to profile the lipids released and depleted by equine embryos. Image created with BioRender.

A visual summary of the experimental procedure, from embryo recovery to mass spectrometry lipid analysis, is presented in Figure 1. Embryos (*n* = 3) were obtained by transcervical uterine lavage 8–9 days after confirmed ovulation, from mares aged between 10 and 11 years, as per the method described by Swegen et al. (29). Briefly, this was done using a 34 French-gage silicone Foley catheter with a 100 cc balloon and Y-tube (MAI Animal Health, Elmwood, WI, United States). For the collection of embryos, warmed Emcare Complete Ultra flushing medium (1000–2000 mL per flush; ICPbio Reproduction, Auckland, New Zealand) was used and uterine fluidic content was collected through an Em-Con embryo filter (MAI Animal Health). Filter contents were transferred to search dishes, and embryos were recovered under a dissecting microscope before being transferred to transport media. This transport medium consisted of Hepes-buffered DMEM/F12 (11330–032; Gibco, Grand Island, NY, United States) supplemented with 0.5% w/v fatty acid-free bovine serum albumin (BSA; ICPbio), 10 units/mL penicillin-G and 10 μg/mL streptomycin sulphate. After transport (<30 min) to the laboratory, each embryo was assessed morphologically and rinsed by moving the embryo through dishes of BSA-containing culture medium (bicarbonate-buffered DMEM/F12 11320–033; Gibco) with 0.5% w/v BSA, 10 units/mL penicillin-G and 10 μg/mL streptomycin sulphate, deposited in a 50 μL droplet of BSA-containing culture medium under oil, and incubated for 2 to 3 h at 38.5°C in a humidified atmosphere of 5% O_2_, 6% CO_2_ and 89% N_2_. Embryos were then washed twice in BSA-free culture medium (bicarbonate-buffered DMEM/F12 with 0.1% polyvinyl alcohol, 10 units/mL penicillin-G and 10 μg/mL streptomycin sulphate). Finally, embryos were transferred to a 50 μL droplet of BSA-free culture medium under oil and incubated at 38.5°C in a humidified atmosphere of 5% O_2_, 6% CO_2_ and 89% N_2_. Following the initial incubation in protein-containing (BSA) medium, embryos were cultured for a total of 36 h in protein-free medium. Within this culture period, embryo-conditioned media were collected every 12 h. At each collection point embryos were imaged under a stereomicroscope (SMZ1500; Nikon Corporation, Kawasaki, Kanagawa, Japan) and their diameter measured to verify continued blastocyst development and expansion. Thereafter embryos washed and transferred to a droplet of fresh BSA-free culture medium before being measured. Embryo-conditioned medium was centrifuged at 14000 × *g* for 5 min to remove debris, and supernatants were transferred to cryovials, and immediately placed in liquid nitrogen, and stored at −80°C until further analysis. Medium-only controls were also collected at each timepoint.

### 2.3 Chemicals and materials

All solvents used were HPLC grade or higher. Glass pipettes and tubes were used wherever possible and the use of plasticware was minimized during lipid extraction to avoid contamination of samples. Glass tubes and glass transfer pipettes were purchased from Sigma and vWR. Lipid internal standards (ISTDs) were purchased from Avanti Polar Lipids Inc. (Alabaster, AL, United States). These include phosphatidylcholine (19:0_19:0), sphingomyelin (18:0_12:0), phosphatidylethanolamine (17:0_17:0), phosphatidylglycerol (17:0_17:0), phosphatidylserine (17:0_17:0), phosphatidic acid (17:0_17:0), ceramide (d18:1, 12:0), diglyceride (1,3 18:0 d5), cholesteryl ester (19:0), monoglyceride (17:0), triglyceride mix d5 (Avanti Code LM-6000), diglyceride mix d5 (Avanti Code LM-6001), phosphatidylinositol (17:0 14:1), C12 GluCer, C12 sulfatide, C17 ceramide, C17 sphingosine, C17 S1P, C12 C1P, D3 C20 fatty acid, and C12 LacCer. Lipid internal standards were prepared as a mixture at 10 pmol/μl in methyl-tert butyl ether and methanol (MTBE:methanol, 1:1 v/v).

### 2.4 Lipid extraction

Lipids were extracted using chloroform, methanol, and isopropanol (Sigma Aldrich, St. Louis, MO, United States) and ultrapure water (Millipore). Medium lipid extraction was based on the Bligh and Dyer method; briefly, medium samples were thawed on ice and 80 μL aliquots were transferred into glass tubes. Methanol (600 μL), chloroform (1,000 μL) and ultrapure water (500 μL) were sequentially added with vortexing between each addition, followed by spiking with 10 μL of the aforementioned internal standards. Samples were then centrifuged at 300 x g for 10 min at room temperature. The lower solvent phase was collected and transferred to a new glass tube using a glass Pasteur pipette. Chloroform (600 μL) was added to the upper phase, vortexed and centrifuged at 900 x g for 10 min. The lower phase was collected and transferred into the same glass tube and dried under nitrogen gas. Dried lipid samples were reconstituted in 100 μL of isopropanol/methanol (1:1) and stored at −80°C in glass LC–MS vials.

### 2.5 Lipidomics mass spectrometry

Lipid extracts (10 μL) were analyzed using a Q-Exactive Plus Mass Spectrometer coupled to a U3000 UPLC system (ThermoFisher Scientific) according to the methods of Phan et al. and Castro-Perez et al. (50, 51). Chromatography was performed at 60°C on a Waters CSH C18 UHPLC column 2.1 × 100 mm, 1.8 μM with VanGuard guard column. Solvent A was 6:4 acetonitrile: water and Solvent B was 1:9 acetonitrile:isopropanol, both with 10 mM ammonium formate and 0.1% formic acid. Briefly, a 30 min gradient running from 30 to 100% of solvent B was performed, eluting lipids in order of hydrophobicity. Column eluate was directed into the electrospray ionization source of the mass spectrometer where a HESI probe was employed. Source parameters were broadly optimized on a range of lipid standards prior to the analysis. The mass spectrometer was run in data dependent acquisition mode. A survey scan over the mass range 200–1,200 at resolution 70 K was followed by 10 data dependent MS/MS scans on the most intense ions in the survey at 15 K resolution. Dynamic exclusion was used to improve the number of ions targeted. Cycle time was approximately 1 s. Samples were run in both positive and negative polarities. The samples were run in a random order (generated using Microsoft Excel) to avoid batch and replicate effects. Data were analyzed in LipidSearch sofware 4.1.16. Data were searched against the standard LipidSearch database with all common mammalian lipid classes included. The search results were then grouped according to sample type and aligned for differential analysis. Aligned data, containing lipid identity, retention time, peak area were exported to Excel software (Microsoft Corporation, WA, United States). Relative abundance of lipids was obtained from peak areas normalized to internal standards. LipidSearch-derived identities of fatty acid chain (fatty acid, FA1, FA2, FA3), CalcMz, IonFormula, retention time (RT) and peak intensity were additionally obtained in both embryo conditioned media (Supplementary Table S1) and in media only experiment (Supplementary Table S2).

### 2.6 Analysis

Identified intensity peaks were filtered with m-Score threshold (>5.0) and ID quality filter (A and B and C). Adducts included +H, +NH_4_, +Na and + H-H_2_O in positive mode, and-H, +HCOO, -2H and-CH3 in negative mode. Statistical analyses were performed using MetaboAnalyst software module^1^ and Excel. For comparisons between time points and control groups, a two tailed students t-test was applied with statistical significance set at *p* < 0.05 in addition to fold changes between embryo-conditioned samples and media only control samples. Graphical representations of the data were generated using the MetaboAnalyst.

### 2.7 Media only samples

In further experiment, the various components of culture media were additionally examined. Different components of culture media were placed in a humidified atmosphere of 5% O_2_, 6% CO_2_ and 89% N_2_ for 12 h at 37°C. Samples were incubated either under a mineral oil overlay or without a mineral oil overlay and included bicarbonate-buffered DMEM/F12 with and without 10 units/mL penicillin-G and 10 μg/mL streptomycin sulphate. Pure mineral oil was also incubated. Incubating media under oil was done to determine whether incubating under oil affected lipid quantification. Thus, a total of five media components/variations were assessed in addition to an ultrapure water (Millipore) in duplicate. Lipids extracted and intensity peaks were analyzed and as per the embryo conditioned media. For the media only samples, ion peaks were compared using one-way analyses to examine whether incubation under oil, DMEM/F12, or penicillin/streptomycin influenced the presence of each lipid in the media. The blocking function was used to account for possible interactions between influencing factors; i.e., analysis for the effect of incubation under oil was ‘blocked’ for presence of DMEM/F12, while analyses for effect of DMEM/F12 and penicillin/streptomycin were ‘blocked’ for incubation under oil to remove the effect of these parameters as confounding factors. Finally, lipid ion peaks in mineral oil were compared against those in all the aqueous media samples (blocking for incubation under oil as a possible confounding factor). These analyses were conducted in JMP (SAS Corp., NC) and *p*-value for all effects set at *p* < 0.05.

## 3 Results

### 3.1 Embryo experiment

**Figure 2:**
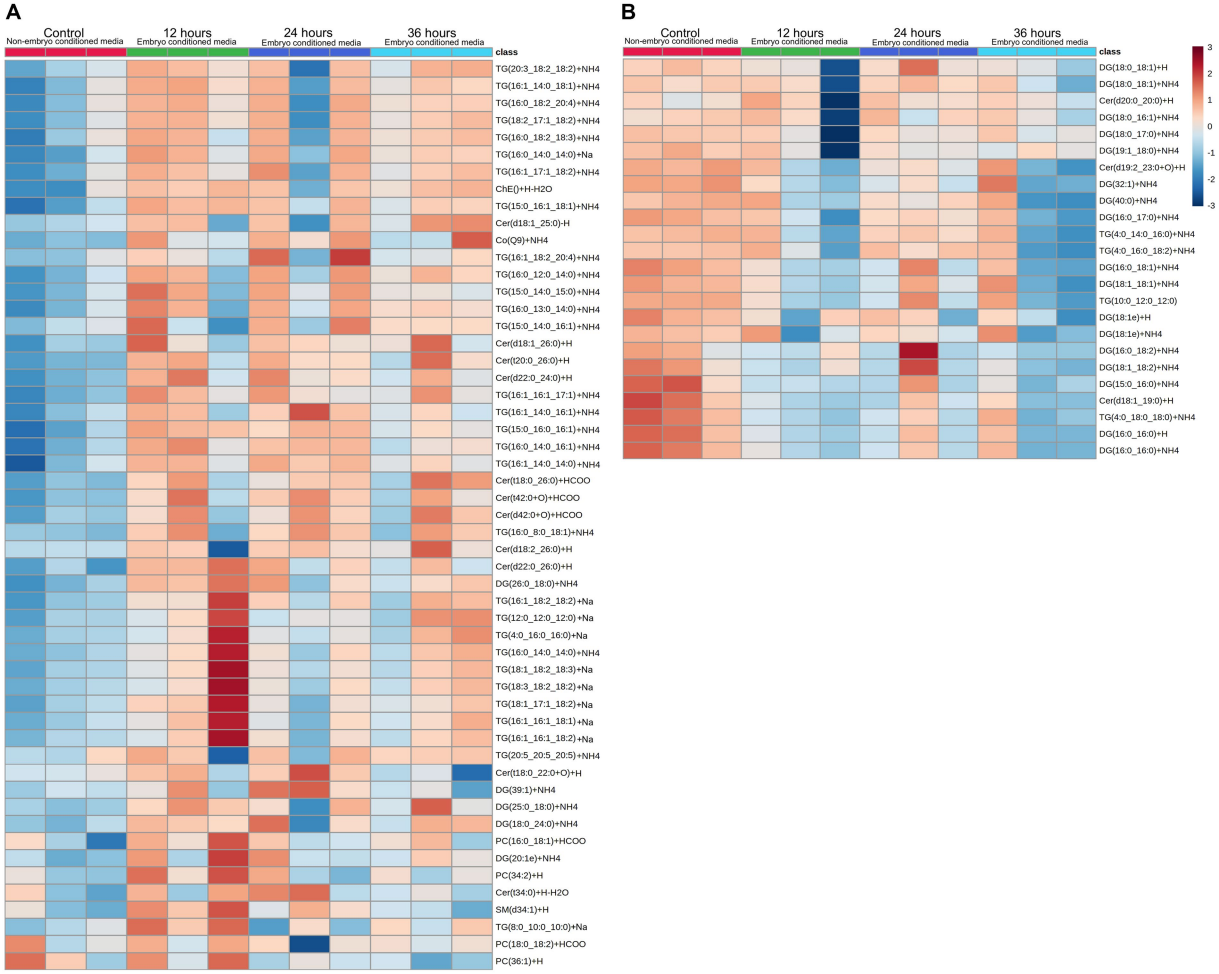
Heatmap visualization of identified lipid abundance (average peak intensity), representing lipid peaks identified. Those secreted **(A)** into embryo culture medium, and those which are depleted **(B)** from the medium. Each column represents the reading from one individual embryo, and each row depicts a different lipid identity. Medium were exchanged every 12 h and embryo-conditioned samples are compared against non-conditioned (embryo-free) control media. The heatmap color scale denotes the relative concentration of each lipid mass relative to the minimum and maximum of that lipid for all groups and is shown on the right-hand side of the figure. Values are measured by Euclidean distance with a Ward clustering algorithm (*n* = 3 per group). *p < 0.05 for each comparison with a fold change of greater than 2.

Embryos (*n* = 3) were cultured *in vitro* in a protein-free medium over a period of 36 h, with each embryo expanding between 20 and 30% in diameter during that time (30.17, 25.40, 20.42%). In the secreted media collected, a total of 1,077 lipids IDs were recorded across all samples, with 222 lipid IDs remaining after filtering for high confidence. Lipids which were both significantly different, in embryo conditioned media versus media only control samples (*p* < 0.05) for at-least one time point, and also had a fold change of greater than 2, were further examined for this study, which was a total of 79 individual lipid ions (Table 1). Of these, 55 individual lipids were detected to be more abundant in embryo-conditioned media (henceforth termed ‘secreted’ lipids), and 24 individual lipids were found to be depleted in embryo-conditioned media (henceforth termed ‘depleted’ lipids). The heatmaps depicted (Figure 2) show two distinct populations of lipids: those that were secreted into the media by the embryos during culture (Figure 2A), and those that were depleted from the media during culture (Figure 2B). Of the 55 lipid identities found elevated in the embryo conditioned media (Table 1), 30 were triacylglycerols (TGs) (54.5%), 13 were ceramides (23.6%), 4 were diacylglycerols (DGs) (7.3%) the sterol lipid cholesterol was also identified along with ubiquinone Co (Q9) and a single sphingomyelin. The 24 lipids found to be depleted from the embryo conditioned media comprised of 17 DGs (70.8%), 4 TGs (16.7%) and 3 ceramides (12.5%). Note that these are the numbers of individual lipid species identified and percentages do not represent quantities of lipid secreted or consumed by embryos.

**Table 1:** Individual lipid species secreted and depleted in embryo-conditioned media according to lipid category.

Result	Lipid species	Category	Percentage according to lipid category
Lipids increased in embryo conditioned media	Cer(d18:1_25:0)-H	Sphingolipids [SP]	25%
	Cer(d18:1_26:0) + H	Sphingolipids [SP]	
	Cer(d18:2_26:0) + H	Sphingolipids [SP]	
	Cer(d22:0_24:0) + H	Sphingolipids [SP]	
	Cer(d22:0_26:0) + H	Sphingolipids [SP]	
	Cer(d42:0 + O) + HCOO	Sphingolipids [SP]	
	Cer(t18:0_22:0 + O) + H	Sphingolipids [SP]	
	Cer(t18:0_26:0) + HCOO	Sphingolipids [SP]	
	Cer(t20:0_26:0) + H	Sphingolipids [SP]	
	Cer(t34:0) + H-H2O	Sphingolipids [SP]	
	Cer(t42:0 + O) + HCOO	Sphingolipids [SP]	
	SM(d34:1) + H	Sphingolipids [SP]	
	ChE() + H-H2O (cholesterol)	Sterol Lipids [ST]	
	Co(Q9) + NH4 (ubiquinone)	Prenol Lipids [PR]	
	DG(18:0_24:0) + NH4	Glycerolipids [GL]	2%
	DG(20:1e) + NH4	Glycerolipids [GL]	2%
	DG(25:0_18:0) + NH4	Glycerolipids [GL]	64%
	DG(26:0_18:0) + NH4	Glycerolipids [GL]	
	DG(39:1) + NH4	Glycerolipids [GL]	
	TG(12:0_12:0_12:0) + Na	Glycerolipids [GL]	
	TG(15:0_14:0_15:0) + NH4	Glycerolipids [GL]	
	TG(15:0_14:0_16:1) + NH4	Glycerolipids [GL]	
	TG(15:0_16:0_16:1) + NH4	Glycerolipids [GL]	
	TG(15:0_16:1_18:1) + NH4	Glycerolipids [GL]	
	TG(16:0_12:0_14:0) + NH4	Glycerolipids [GL]	
	TG(16:0_13:0_14:0) + NH4	Glycerolipids [GL]	
	TG(16:0_14:0_14:0) + Na	Glycerolipids [GL]	
	TG(16:0_14:0_14:0) + NH4	Glycerolipids [GL]	
	TG(16:0_14:0_16:1) + NH4	Glycerolipids [GL]	
	TG(16:0_18:2_18:3) + NH4	Glycerolipids [GL]	
	TG(16:0_18:2_20:4) + NH4	Glycerolipids [GL]	
	TG(16:0_8:0_18:1) + NH4	Glycerolipids [GL]	
	TG(16:1_14:0_14:0) + NH4	Glycerolipids [GL]	
	TG(16:1_14:0_16:1) + NH4	Glycerolipids [GL]	
	TG(16:1_14:0_18:1) + NH4	Glycerolipids [GL]	
	TG(16:1_16:1_17:1) + NH4	Glycerolipids [GL]	
	TG(16:1_16:1_18:1) + Na	Glycerolipids [GL]	
	TG(16:1_16:1_18:2) + Na	Glycerolipids [GL]	
	TG(16:1_17:1_18:2) + NH4	Glycerolipids [GL]	
	TG(16:1_18:2_18:2) + Na	Glycerolipids [GL]	
	TG(16:1_18:2_20:4) + NH4	Glycerolipids [GL]	
	TG(18:1_17:1_18:2) + Na	Glycerolipids [GL]	
	TG(18:1_18:2_18:3) + Na	Glycerolipids [GL]	
	TG(18:2_17:1_18:2) + NH4	Glycerolipids [GL]	
	TG(18:3_18:2_18:2) + Na	Glycerolipids [GL]	
	TG(20:3_18:2_18:2) + NH4	Glycerolipids [GL]	
	TG(20:5_20:5_20:5) + NH4	Glycerolipids [GL]	
	TG(4:0_16:0_16:0) + Na	Glycerolipids [GL]	
	TG(8:0_10:0_10:0) + Na	Glycerolipids [GL]	
	PC(16:0_18:1) + HCOO	Glycerophospholipids [GP]	
	PC(18:0_18:2) + HCOO	Glycerophospholipids [GP]	
	PC(34:2) + H	Glycerophospholipids [GP]	7%
	PC(36:1) + H	Glycerophospholipids [GP]	
	Cer(d18:1_19:0) + H	Sphingolipids [SP]	
	Cer(d19:2_23:0 + O) + H	Sphingolipids [SP]	
Lipids depleted in embryo conditioned media	Cer(d20:0_20:0) + H	Sphingolipids [SP]	13%
	DG(15:0_16:0) + NH4	Glycerolipids [GL]	
	DG(16:0_16:0) + H	Glycerolipids [GL]	
	DG(16:0_16:0) + NH4	Glycerolipids [GL]	88%
	DG(16:0_17:0) + NH4	Glycerolipids [GL]	
	DG(16:0_18:1) + NH4	Glycerolipids [GL]	
	DG(16:0_18:2) + NH4	Glycerolipids [GL]	
	DG(18:0_16:1) + NH4	Glycerolipids [GL]	
	DG(18:0_17:0) + NH4	Glycerolipids [GL]	
	DG(18:0_18:1) + H	Glycerolipids [GL]	
	DG(18:0_18:1) + NH4	Glycerolipids [GL]	
	DG(18:1_18:1) + NH4	Glycerolipids [GL]	
	DG(18:1_18:2) + NH4	Glycerolipids [GL]	
	DG(18:1e) + H	Glycerolipids [GL]	
	DG(18:1e) + NH4	Glycerolipids [GL]	
	DG(19:1_18:0) + NH4	Glycerolipids [GL]	
	DG(32:1) + NH4	Glycerolipids [GL]	
	DG(40:0) + NH4	Glycerolipids [GL]	
	TG(4:0_14:0_16:0) + NH4	Glycerolipids [GL]	
	TG(4:0_16:0_18:2) + NH4	Glycerolipids [GL]	
	TG(4:0_18:0_18:0) + NH4	Glycerolipids [GL]	
	TG(10:0_12:0_12:0) + NH4	Glycerolipids [GL]	
	TG(10:0_12:0_12:0) + NH4	Glycerolipids [GL]	
	TG(10:0_12:0_12:0) + NH4	Glycerolipids [GL]	

**Figure 3:**
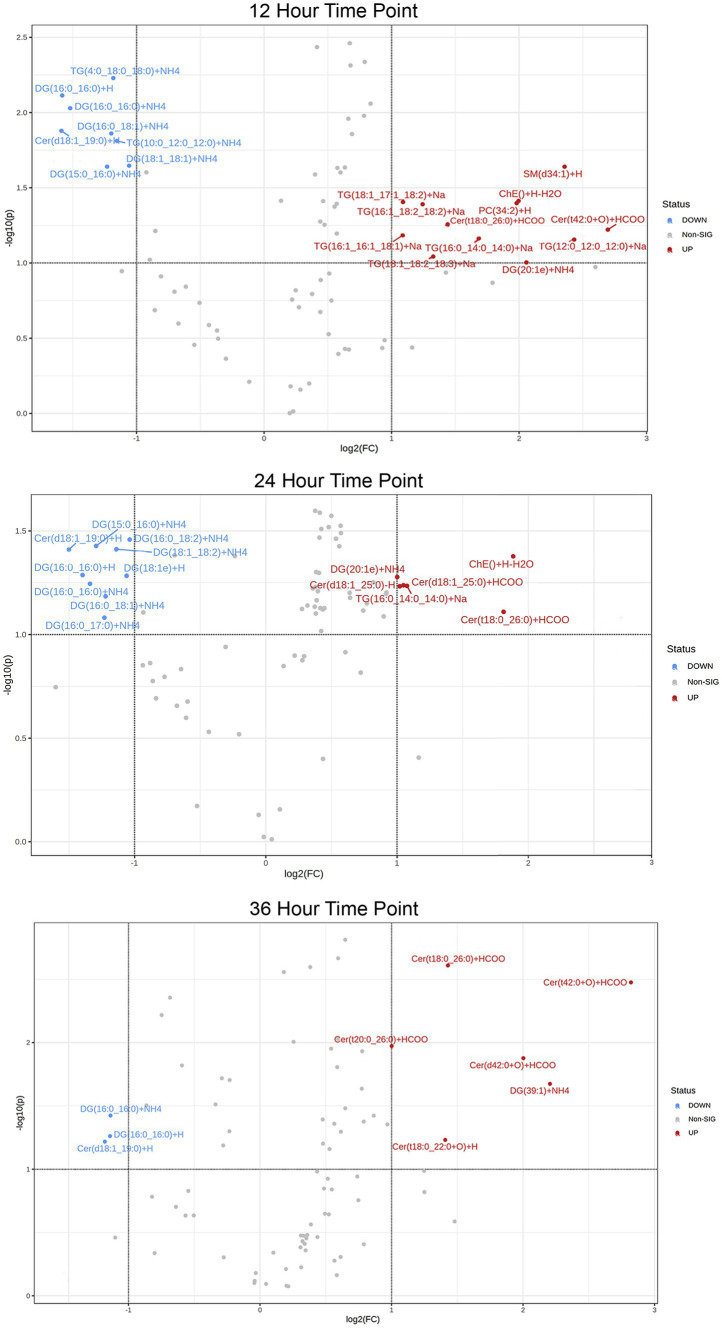
Volcano plot of lipid IDs from embryo secreted (red) and depleted (blue) medium. On the y-axis lipids with *p* < 0.1 are shown, with fold change (FC) >2 depicted on the x-axis and significance on y-axis.

**Figure 4:**
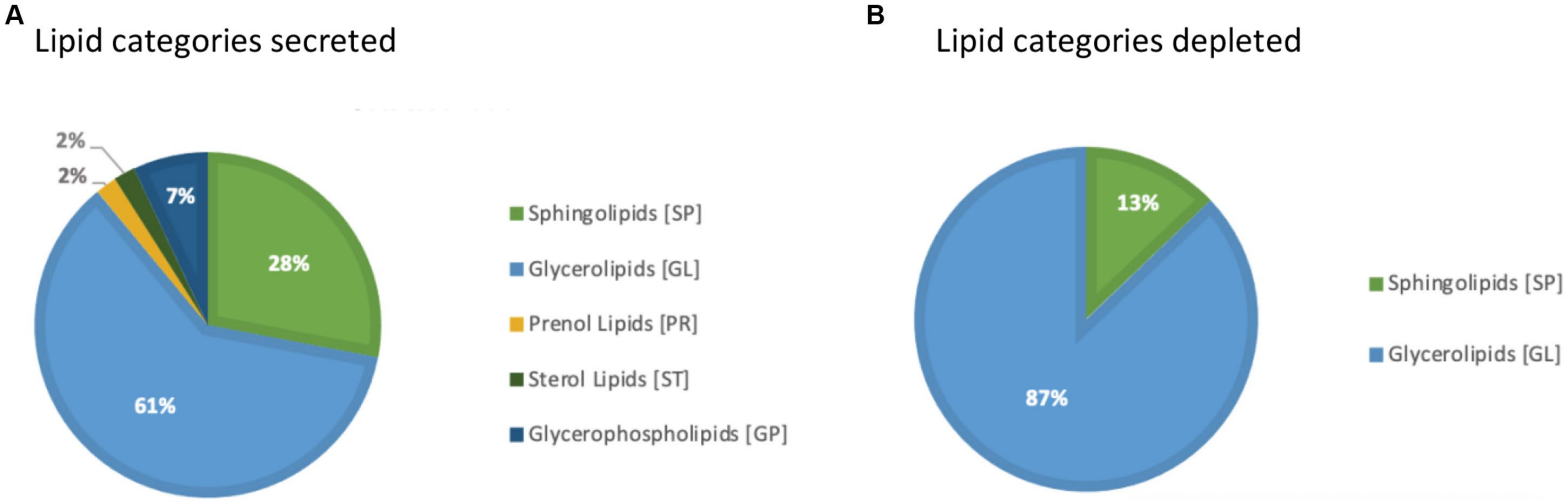
Pie graph of lipid categories secreted **(A)** and depleted **(B)** in embryo-conditioned media.

**Figure 5:**
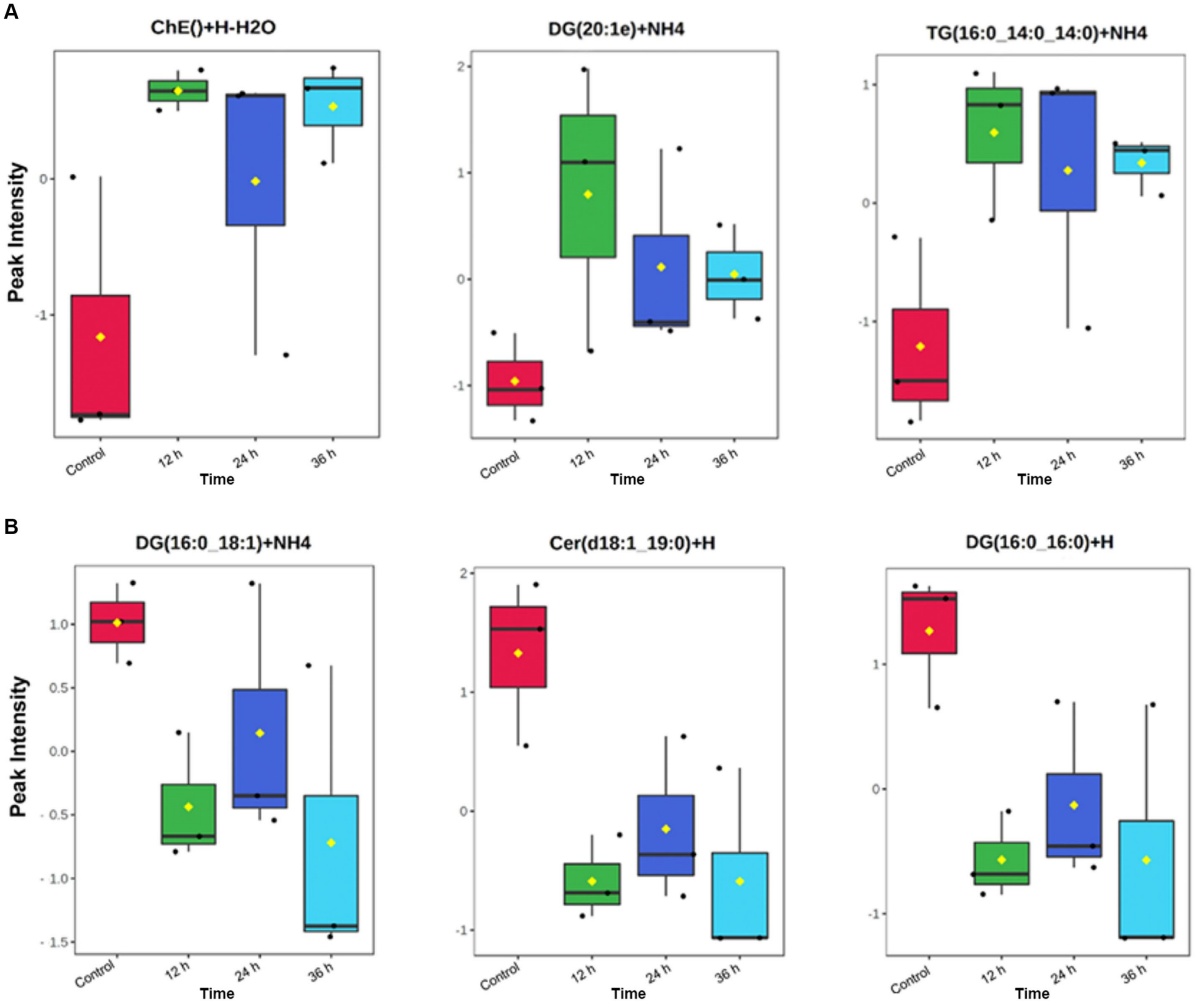
Individual identified lipids secreted **(A)** and depleted **(B)** in embryo-conditioned media. Representations of the intensity peaks for individual species with box and whisker plots. Each individual lipid is both significantly different between time points and has a fold has a fold change >2. Axis Y: Normalized peak intensities (areas) shown.

In addition, a volcano plot analysis was carried out to pinpoint the differential change of individual lipids between the groups, with a cut-off for those lipids that had fold-change >2 and *p*-value <0.1. This was done for each individual time point, 12 h, 24 h and 36 h. In the embryo secreted group, ceramides and TGs were the predominant classes found to increase compared to the control. From the depleted lipid group, DGs were observed to be the dominant class (Figure 3). In total, the classes of lipids came from 5 lipid categories, including glycerolipids, sphingolipids, glycerophospholipids, sterol lipids and prenol lipids (Figure 4), with a greater diversity of classes observed in secreted lipids compared to depleted lipids. Comparisons of several individual relevant lipids, which were both significant and had a fold change >2 in the embryo conditioned media, are detailed in Figure 5.

### 3.2 Culture media only experiment

Lipid-free reagents had been used to prepare media for embryo lipidome experiments, so the detection of lipids depleted following embryo culture was unexpected. We therefore conducted a follow-up analysis several months later to validate the presence of lipids in non-conditioned (control) media and its individual components. For the media only experiment, a total of 137 lipid species were identified present across all 5 media samples (DMEM/F12 with and without 10 units/mL penicillin-G and 10 μg/mL streptomycin sulphate, both of these under a mineral oil overlay or without a mineral oil overlay and lastly pure mineral oil). These predominantly belong to the glycerolipid category. Of these, 109 lipid ions were significantly different between groups. Higher abundance of 18 lipids (15 TGs, 2 DGs, 1 PS) was associated with inclusion of DMEM/F12 in the formulation (predominantly glycerolipids; 94%). The inclusion of penicillin-G/streptomycin sulphate to DMEM/F12 medium had no influence on abundance of any of the lipids detected. Incubation under oil increased the abundance of 44 lipid species in media (90% were glycerolipids; 34 TGs and 5 DGs). In pure mineral oil, 47 lipid species were detected (i.e., showed *p* < 0.05 versus water only control). Of these, 91% were glycerolipids (28 TGs and 15 DGs), and 6% (3) were ceramides. Of the 24 DGs originally found to be depleted in embryo-conditioned media, promisingly only 5 indivuiduals were detected in the follow-up media analysis. Of these, two lipids were detected in mineral oil alone and were influenced by incubation under mineral oil [DG (18:0_17:0) + NH4 and DG(18:0_18:1) + NH4]; one lipid was detected in mineral oil but not affected by incubation under oil [DG(40:0) + NH4].

## 4 Discussion

This study used high-resolution mass spectrometry-based lipidomics to examine the changes in lipid profiles of media following culture of equine embryos. A total of 55 lipid species were found to be significantly increased in the embryo-conditioned media, compared to the control, and hence were presumed to be released or secreted by the embryo over time. In addition, 24 lipids were identified to be significantly decreased in the embryo-conditioned media following embryo incubation when compared to the embryo-free controls. These lipids were presumed to be taken up or depleted from the media by the embryos. After identifying this population of depleted lipids, a more comprehensive analysis was carried out of media without embryos. This was conducted to clarify which lipids or lipid categories were detectable in culture media, and in turn which components of the culture environment they were coming from. In this subsequent study we found that 109 lipids were contributed by the various media components, the majority of these being glycerolipids.

For those lipids found to be increased in the embryo-conditioned media, the dominant lipid categories identified were glycerolipids (66%) followed by sphingolipid (23%); the dominant lipid classes were TGs (56.6%) and ceramides (20.8%). Those depleted from the media in the presence of embryos again tended to be glycerolipids (88%), with the main class of these found to be DGs (70.8%). The depleted lipid groups May contribute to meeting the embryo’s energy demands, whereas the secreted lipid groups are likely to either be involved in maternal signaling, or to be released in response to stress/increased bilayer fluidity. TGs are esters consisting of a glycerol backbone and three fatty acids; they represent the main form of lipid storage in adipose tissue and lipid droplets. TGs are synthesized in times of energy excess or hydrolyzed to DGs and fatty acids to be used for ATP generation in times of energy need. Thus, from a metabolic perspective, release of TGs by equine embryos indicates that energy stores May be adequate or excessive. Closer examination of the exact identities of TGs being secreted will be important in elucidating their potential roles in embryo-maternal signaling. Most of the secreted TGs that were identified contain medium-chain fatty acids. Medium chain fatty acids have been shown to enhance progesterone synthesis and improve embryo implantation in rats, albeit via a mechanism of dietary supplementation of fatty acids to the mother (52). It is worth investigating the direct effects of medium chain fatty acid TGs on the endometrium and whether their secretion by the embryo is able to influence pathways upstream of luteinisation and luteal maintenance, such as reduced PGF2a synthesis. The product of TG hydrolysis, DGs, consist of a glycerol covalently bonded to two fatty acid chains. They are typically found in both plant and animal fats, and are often used as emulsifiers (53). DGs are not only metabolic substrates but can activate certain lipid-sensitive receptors. Cellular DGs can bind to members of the protein kinase C (PKC) family, which leads to their activation and translocation to the plasma membrane and subsequent phosphorylation of interacting proteins (54). PKCs have been identified in developing embryos, and PKC inhibition halts embryo development beyond the eight to 16-cell stage in bovine embryos (55). Considering these observations, it is plausible that DGs in embryo culture media or mare uterine fluid have the capacity to support embryo development both as an energy source and as a messenger system that activates developmental processes via receptor activation. As DGs are metabolized by the lipase pathway for prostaglandin synthesis, while prostaglandins are suspected to stimulate the myometrial contractions that propel the conceptus throughout the uterine lumen during days 10–16 after ovulation (39, 56). If equine embryos do indeed actively deplete DGs from their direct environment, this May be related to their known ability to produce and continue to secrete prostaglandin E2 and other prostaglandins (39).

**Figure 6:**
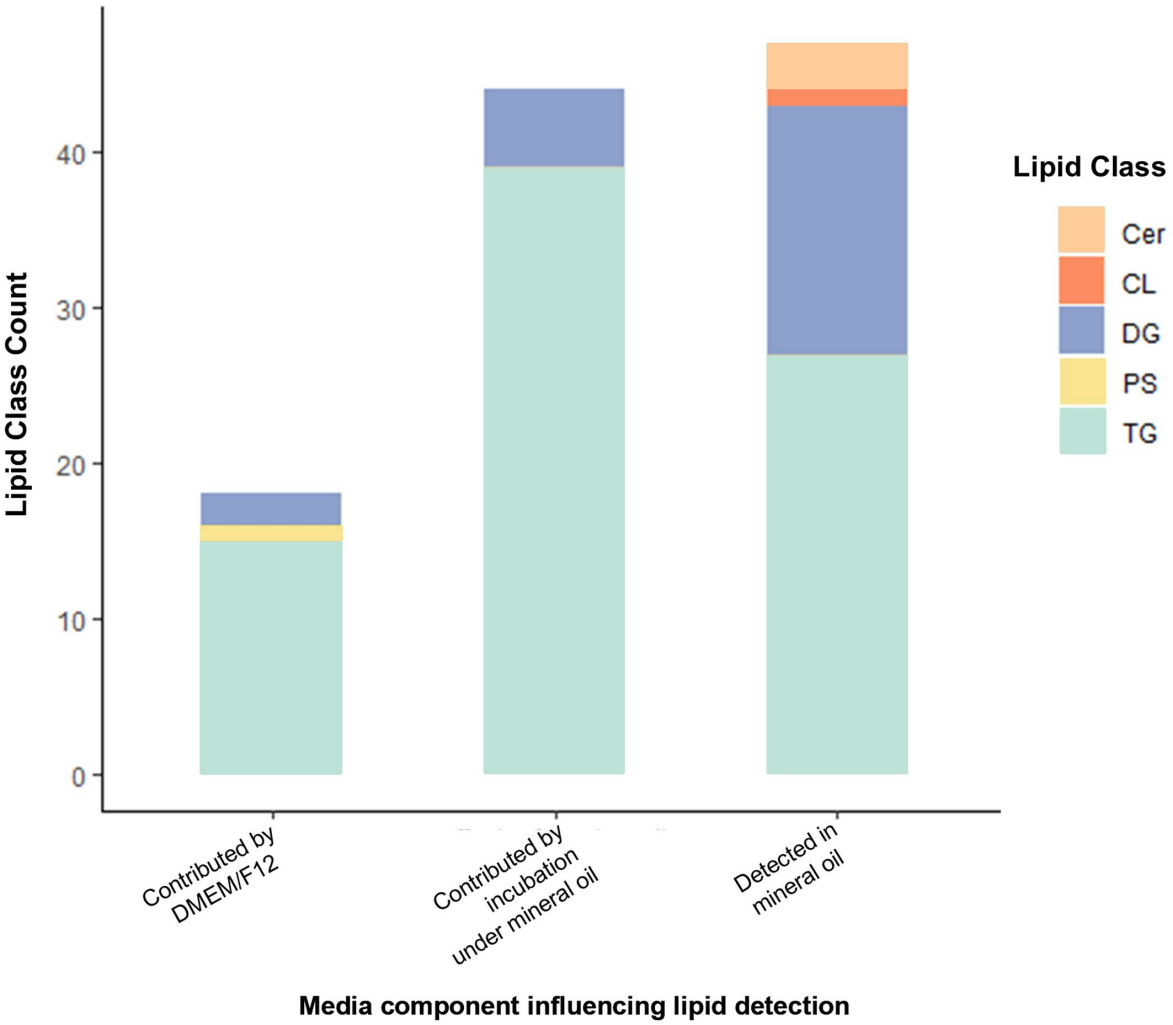
Lipid IDs whose detection was influenced by inclusion of DMEM/F12 or by incubation under mineral oil, and those detected in mineral oil. Cer, Ceramide; CL, Cardiolipin; DG, Diacylglycerol; PS, Phosphatidylserine; TG, Triacylglycerols. All lipids shown are those with *p* < 0.05 contribution from media components indicated (as determined by one-way analysis).

In this study most importantly, several individual lipids were both significantly increased and had a fold change of at least 2 in the embryo conditioned media (Figure 6). The sterol lipid cholesterol increased across all time points, including an approximate 8-fold increase at 12 h. In reproduction, the role of cholesterol is essential in early conceptus development as it maintains the integrity/fluidity of cell membranes and plays an important role in cell signaling (57). The lipid moderates important nuclear receptors, such as fetoprotein transcription factor, and is therefore involved in the most fundamental signaling pathways during embryonic development (58). Cholesterol is also the precursor of all steroid hormones (59) from which both progesterone and oestradiol are synthesized through the precursor steroid, pregnenolone. As such, the detection of cholesterol is particularly pertinent as oestradiol production by the early equine conceptus is considered very significant to the establishment of pregnancy (60) with substantial quantities of estrogens known to be produced by day 12 equine conceptus (61, 62). Another individual lipid, Ubiquinone Co(Q9), which is categorized as a prenol lipid (63) was significantly elevated in the media across all time points (Figure 6). Co(Q9) is an essential component of the mitochondrial electron transfer chain and thus is required for ATP synthesis. The presence of such lipids and their subsequent increase over time is encouraging, as they indicate embryos are producing a lipid profile that is bioactivity relevant. The cascade of events that occurs around the time of MRP indicates that lipids, such as Cholesterol, May play a more important role in the establishment of pregnancy both in terms of what the embryo is synthesizing and how the maternal endometrium is changing in response to such precursors.

For investigations into mare MRP, studies have examined the endometrial gene expression changes in response to the presence of a conceptus during MRP (64–68). Genes involved in lipid metabolism and those that serve a lipid biosynthetic process were found to be upregulated and overrepresented in the luminal epithelium of early pregnant mares (69). The prostaglandin transporter gene SLCO2A1, changes cyclically during the menstrual cycle in the human endometrium. In the bovine early embryo (70) and porcine early embryo (71, 72), its expression has been reported to increase. This gene was also found to be upregulated within the luminal epithelium of the pregnant mare (73), highlighting the importance of lipid-associated gene expression around the time of MRP. Interestingly, many of the genes upregulated in endometrial epithelium are involved in sphingolipid metabolism and signaling. The sphingolipid ceramide is known to be involved in regulation of proliferation via its signaling role (74), and thus embryo-secreted ceramides, such as those found in this study, could form part of the embryo-maternal signaling mechanism that stimulates or regulates proliferation of endometrial glands, which support the embryo’s nutritional demands. Interestingly, ceramides can mimic the actions of progesterone in inducing maturation of amphibian oocytes *in vitro*, via the non-classical (membrane) progesterone receptor (75). Given the central role progesterone plays in supporting the early pregnancy, the influence of ceramides during *in vitro* culture requires further attention.

While we initially set out to profile the lipids increased in the media by the presence of equine embryos, the results of this study demonstrate that the “lipid-free” media contained consistently detectable amounts of lipids. The detection of MS peaks of residual lipids in the control media samples was an unexpected finding; some of these were significantly lower in the embryo-conditioned media than control media. The additional analysis of non-conditioned medium confirmed the presence of numerous glycerolipids in embryo-free culture media, the majority of which were DGs and TGs. Furthermore, lipid peaks in non-conditioned medium increased in response to culture under mineral oil, while mineral oil itself evidently contained ceramides, DGs and TGs, these were surprising findings. To our knowledge, the alteration of culture medium lipid composition by mineral oil overlay has not been described previously. Given the influence of lipids on preimplantation embryo development, the effect of culturing embryos in medium with and without an oil overlay warrants additional investigation. As this is the first lipidomic study of the embryo secretome in mammals, the identification of potential glycerolipid uptake by embryos is of relevance to all mammalian embryos cultured *in vitro*. The definitive origin of these lipids remains uncertain but DMEM/F12 and mineral oil could not be ruled out as a potential source. Other possible origins include, but are not limited to, pipette tips, petri dishes, cryovials, streptomycin, or the ever-present possibility of sample contamination during handling. We propose that the predominant source of unknown lipids in our media was transfer from the fresh mineral oil. Furthermore, the identification of this lipid population highlights the capacity of the technology to detect and identify minute amounts of lipids in samples.

As reported in animal models, the lipid composition of culture medium can disturb embryo metabolism and gene expression and have consequences on preimplantation development and the health of resulting offspring following embryo transfer (76, 77). The presence and uptake of an unspecified population of lipids May indeed influence future development. As the embryo requirements for lipids during *in vitro* culture are not well understood, this area of enquiry has gained increasing attention over the past decade. Studies in which culture media were supplemented with different quantities and or different types of lipids have shown a range of effects on embryo development in various species (78). Notably the mare has recently gained traction as a particularly suitable model for investigations of human fertility and pregnancy, given it is a mono-ovulatory mammal with a gestation of a similar duration, as well as pertinent similarities in embryo development trajectory and mechanisms of pregnancy loss (7, 79, 80). In humans, different commercial embryo culture media have been reported to alter embryo quality and influence birthweight, with one study finding that supplementing embryo culture media with Human Serum Albumin (HSA) that contained high levels of residual lipids correlated with poorer pregnancy and fetal outcomes (81). The type of lipid is of great importance, as illustrated by supplementation of embryo culture media with saturated fatty acids, which was associated with metabolic stress, developmental delays, increased ROS production, and reduced implantation rates (81, 82). In addition, in the context of maternal communication, the ability for extracellular vesicles (EVs) to transfer molecular cargo, such as lipids, from one cell to another has generated a growing interest (83), and is relevant for our findings. Embryo-derived EVs have been pointed to as modulators of embryo communication *in vitro* (84), and embryos cultured *in vitro* have been confirmed to secrete EVs into their immediate environment (84, 85). Such research suggests that lipids and lipid-containing molecule, such as liposomes May also be secreted in the form of EVs by equine embryos, and hence the role of EVs and lipid transfer through media warrants further investigation.

In acknowledging the limitations of this study, the small sample size must be noted, along with the potential for the *in vitro* environment to induce a stress response. The *in vitro* culture conditions did support continued embryo growth and survival in this study, but it would still be prudent to assume that the change in environment elicited some stress in the embryos. Therefore, we do not know whether the ceramide secretion seen here is physiological or a response to the stress of *in vitro* culture. In liver, skeletal muscle, heart, kidney, and pancreatic *β* cells, elevated ceramide secretion has been observed in response to cellular stress and lipotoxicity, leading to apoptosis (86–88). Experiments examining the direct effects of ceramides on the endometrium will help to determine if these molecules play a role in embryo-maternal signaling. Lipotoxicity seems an unlikely cause for ceramide release in the present study, since the medium used for embryo culture was essentially lipid-free, even though residual levels of lipids were detected in the medium-only controls. Nevertheless, it must be considered that early embryos May be very sensitive to the lipid milieu and the precise requirements and sensitivities of the mammalian embryo need to be better understood, especially with regard to lipids in the immediate embryonic environment. Of note is that phytoceramides (tCer) are much less abundant in mammalian cells than in yeast (89) and are predominantly recovered in plants (90) a few of which were identified in the current data by LipidSearch, using diagnostic fragment ions (with all common mammalian lipid classes included). So, although mammalian cells do contain phytoceramides (91, 92), the subcellular localisation of their synthesis is still a matter of debate. As with any emerging technology, the field of lipidomics is still being refined. Further confirmatory work with targeted LC–MS analysis utilizing authentic standards, would be helpful to confirm whether these are secreted phytoceramides or mislabelled by the software. Thus, despite not being abundant in mammals, the identification of tCers warrants both inclusion in these findings, and future deeper investigation.

To summarize, this study presents the first high-resolution lipidomic profile of the pre-implantation embryo secretome in any mammalian species. Predominantly glycerolipids, and in particular triglycerides, were consistently released by early equine embryos into their culture environment. While the precise functions of defined lipids remain to be investigated, many of the lipids identified in this study have documented roles in cell signaling, metabolism and embryo development. Meanwhile, a cohort of lipids were depleted from media during culture, predominantly diglycerides. Since diglycerides have a documented capacity to regulate embryo developmental processes, these findings emphasize the importance of a defined embryo culture medium and a need to identify the lipid requirements of the embryo precisely. We also highlight that culture media formulated as “lipid-free” contains detectable amounts of a wide range of lipid species, many of which are absorbed by the early embryo. The profiles described contribute new knowledge about early embryo lipid metabolism and production during a critical period of pre-implantation development, which will contribute to our understanding of early equine pregnancy. Further investigation of the specific lipid ions identified in this study can clarify their functional roles in early equine pregnancy as well as the uptake of lipids present in the embryo culture environment.

## Data Availability

The original contributions presented in the study are included in the article/Supplementary material, further inquiries can be directed to the corresponding author/s.

## Footnotes

^1^ www.metaboanalyst.ca

## Supplementary material

Supplementary Table S1 Full dataset of lipid ions detected in embryo-conditioned culture media.

Supplementary Table S2 Full dataset of lipid ions detected in ‘media only’ experiment.

## References

[1] Jarvis GE. Early embryo mortality in natural human reproduction: what the data say. F1000Res. (2016) 5:2765. doi: 10.12688/f1000research.8937.1 28580126 PMC5443340

[2] Satué K Gardon JC. Pregnancy loss in mares In: Atef MD, editor. Genital infections and infertility. Rijeka: IntechOpen (2016)

[3] Vanderwall DK. Early embryonic loss in the Mare. J Equine Vet. (2008) 28:691–702. doi: 10.1016/j.jevs.2008.10.001

[4] Stout TA. Embryo-maternal communication during the first 4 weeks of equine pregnancy. Theriogenology. (2016) 86:349–54. doi: 10.1016/j.theriogenology.2016.04.048, PMID: 27156682

[5] Swegen A. Maternal recognition of pregnancy in the mare: does it exist and why do we care? Reproduction. (2021) 161:R139–r155. doi: 10.1530/REP-20-0437, PMID: 33957605 PMC8183633

[6] Salamonsen LA Evans J Nguyen HP Edgell TA. The microenvironment of human implantation: determinant of reproductive success. Am J Reprod Immunol. (2016) 75:218–25. doi: 10.1111/aji.12450, PMID: 26661899

[7] Benammar A Derisoud E Vialard F Palmer E Ayoubi JM Poulain M. The Mare: A pertinent model for human assisted reproductive technologies? Animals (Basel). (2021) 11:2304. doi: 10.3390/ani11082304 34438761 PMC8388489

[8] Carnevale E Ginther O. Relationships of age to uterine function and reproductive efficiency in mares. Theriogenology. (1992) 37:1101–15. doi: 10.1016/0093-691X(92)90108-4, PMID: 16727108

[9] Cuervo-Arango J Claes AN Stout TA. A retrospective comparison of the efficiency of different assisted reproductive techniques in the horse, emphasizing the impact of maternal age. Theriogenology. (2019) 132:36–44. doi: 10.1016/j.theriogenology.2019.04.010, PMID: 30986613

[10] Fretts RC Schmittdiel J Mclean FH Usher RH Goldman MB. Increased maternal age and the risk of fetal death. N Engl J Med. (1995) 333:953–7. doi: 10.1056/NEJM199510123331501 7666913

[11] Gonzalez MB Robker RL Rose RD. Obesity and oocyte quality: significant implications for ART and emerging mechanistic insights. Biol Reprod. (2022) 106:338–50. doi: 10.1093/biolre/ioab228, PMID: 34918035

[12] Sessions-Bresnahan DR Schauer KL Heuberger AL Carnevale EM. Effect of obesity on the Preovulatory follicle and lipid fingerprint of equine oocytes. Biol Reprod. (2016) 94:15. doi: 10.1095/biolreprod.115.130187 26632608

[13] Catandi GD Obeidat YM Broeckling CD Chen TW Chicco AJ Carnevale EM. Equine maternal aging affects oocyte lipid content, metabolic function and developmental potential. Reproduction. (2021) 161:399–409. doi: 10.1530/REP-20-0494, PMID: 33539317 PMC7969451

[14] Lawson EF Grupen CG Baker MA Aitken RJ Swegen A Pollard C-L. Conception and early pregnancy in the mare: lipidomics the unexplored frontier. Reprod Fertil. (2022) 3:R1–R18. doi: 10.1530/RAF-21-0104, PMID: 35350651 PMC8956829

[15] Abe H Yamashita S Satoh T Hoshi H. Accumulation of cytoplasmic lipid droplets in bovine embryos and cryotolerance of embryos developed in different culture systems using serum-free or serum-containing media. Mol Reprod Dev. (2002) 61:57–66. doi: 10.1002/mrd.1131 11774376

[16] Foss R Ortis H Hinrichs K. Effect of potential oocyte transport protocols on blastocyst rates after intracytoplasmic sperm injection in the horse. Equine Vet J. (2013) 45:39–43. doi: 10.1111/evj.12159, PMID: 24304402

[17] Wale PL Gardner DK. The effects of chemical and physical factors on mammalian embryo culture and their importance for the practice of assisted human reproduction. Hum Reprod Update. (2016) 22:2–22. doi: 10.1093/humupd/dmv034, PMID: 26207016

[18] Yu K Pfeiffer C Burden C Krekeler N Marth C. High ambient temperature and humidity associated with early embryonic loss after embryo transfer in mares. Theriogenology. (2022) 188:37–42. doi: 10.1016/j.theriogenology.2022.05.014, PMID: 35661481

[19] Pickering SJ Johnson MH Braude PR Houliston E. Cytoskeletal organization in fresh, aged and spontaneously activated human oocytes. Hum Reprod. (1988) 3:978–89. doi: 10.1093/oxfordjournals.humrep.a136828, PMID: 3204153

[20] Wang W-H Sun Q-Y. Meiotic spindle, spindle checkpoint and embryonic aneuploidy. Front Biosci. (2006) 11:620–36. doi: 10.2741/1822 16146756

[21] Felix MR Turner RM Dobbie T Hinrichs K. Successful in vitro fertilization in the horse: production of blastocysts and birth of foals after prolonged sperm incubation for capacitation†. Biol Reprod. (2022) 107:1551–64. doi: 10.1093/biolre/ioac172, PMID: 36106756 PMC9752964

[22] Leemans B Gadella BM Stout TA De Schauwer C Nelis H Hoogewijs M. Why doesn't conventional IVF work in the horse? The equine oviduct as a microenvironment for capacitation/fertilization. Reproduction. (2016) 152:R233–r245. doi: 10.1530/REP-16-0420 PMID: 27651517

[23] Gross RW Han X. Lipidomics at the interface of structure and function in systems biology. Chem Biol. (2011) 18:284–91. doi: 10.1016/j.chembiol.2011.01.014, PMID: 21439472 PMC3132894

[24] Saliba AE Vonkova I Gavin AC. The systematic analysis of protein-lipid interactions comes of age. Nat Rev Mol Cell Biol. (2015) 16:753–61. doi: 10.1038/nrm4080, PMID: 26507169

[25] Hatzel JN Bouma GJ Cleys ER Bemis LT Ehrhart EJ Mccue PM. Identification of heat shock protein 10 within the equine embryo, endometrium, and maternal peripheral blood mononuclear cells. Theriogenology. (2015) 83:832–9. doi: 10.1016/j.theriogenology.2014.11.020, PMID: 25542459

[26] Lawson EF Gibb Z De Ruijter-Villani M Smith ND Stout TA Clutton-Brock A. Proteomic analysis of pregnant Mare uterine fluid. J Equine Vet. (2018) 66:171–2. doi: 10.1016/j.jevs.2018.05.064

[27] Scholtz E Ball B Stanley S Moeller B Conley A. *Bioactivity of 5α-dihydroprogesterone in mares: endometrial response and maintenance of early pregnancy*. Proceedings of the 55th Annual Convention of the American Association of Equine Practitioners, Las Vegas, Nevada, USA, American Association of Equine Practitioners (AAEP), 262–263. (2009).

[28] Smits K Nelis H Van Steendam K Govaere J Roels K Ververs C. Proteome of equine oviducal fluid: effects of ovulation and pregnancy. Reprod Fertil Dev. (2017) 29:1085–95. doi: 10.1071/RD15481, PMID: 27120206

[29] Swegen A Grupen CG Gibb Z Baker MA De Ruijter-Villani M Smith ND. From peptide masses to pregnancy maintenance: A comprehensive proteomic analysis of the early equine embryo Secretome, blastocoel fluid, and capsule. Proteomics. (2017) 17:1600433. doi: 10.1002/pmic.201600433, PMID: 28782881

[30] Klein C Troedsson MH. Transcriptional profiling of equine conceptuses reveals new aspects of embryo-maternal communication in the horse. Biol Reprod. (2011) 84:872–85. doi: 10.1095/biolreprod.110.088732 21209420

[31] Ohnuma K Yokoo M Ito K Takahashi J Nambo Y Miyake YI. Study of early pregnancy factor (EPF) in equine (*Equus caballus*). Am J Reprod Immunol. (2000) 43:174–9. doi: 10.1111/j.8755-8920.2000.430307.x, PMID: 10735594

[32] Lawson EF Ghosh A Grupen C Netherton J Aitken RJ Smith ND. Investigations into the role of platelet-activating factor in the peri-conception period of the mare. Reproduction. (2024) 168:e240049. doi: 10.1530/REP-24-0049 39056485

[33] O’Neill C. The role of paf in embryo physiology. Hum Reprod Update. (2005) 11:215–28. doi: 10.1093/humupd/dmi003 15790601

[34] Dutta-Roy AK. Fatty acid transport and metabolism in the feto-placental unit and the role of fatty acid-binding proteins. J Nutr Biochem. (1997) 8:548–57. doi: 10.1016/S0955-2863(97)00087-9

[35] Stout TA Meadows S Allen WR. Stage-specific formation of the equine blastocyst capsule is instrumental to hatching and to embryonic survival in vivo. Anim Reprod Sci. (2005) 87:269–81. doi: 10.1016/j.anireprosci.2004.11.009, PMID: 15911176

[36] Oriol JG Sharom FJ Betteridge KJ. Developmentally regulated changes in the glycoproteins of the equine embryonic capsule. J Reprod Fertil. (1993) 99:653–64. doi: 10.1530/jrf.0.0990653, PMID: 8107051

[37] Flood PF Betteridge KJ Diocee MS. Transmission electron microscopy of horse embryos 3-16 days after ovulation. J Reprod Fertil Suppl. (1982) 32:319–27. PMID: 6962867

[38] Gibson C De Ruijter-Villani M Rietveld J Stout TAE. Expression of glucose transporters in the endometrium and early conceptus membranes of the horse. Placenta. (2018) 68:23–32. doi: 10.1016/j.placenta.2018.06.308, PMID: 30055666

[39] Stout TA Allen WR. Prostaglandin E(2) and F(2 alpha) production by equine conceptuses and concentrations in conceptus fluids and uterine flushings recovered from early pregnant and dioestrous mares. Reproduction. (2002) 123:261–8. doi: 10.1530/rep.0.1230261, PMID: 11866693

[40] Weber J Woods G Freeman D Vanderwall D. Prostaglandin E2-specific binding to the equine oviduct. Prostaglandins. (1992) 43:61–5. doi: 10.1016/0090-6980(92)90065-2, PMID: 1546174

[41] Weber JA Freeman DA Vanderwall DK Woods GL. Prostaglandin E2 secretion by oviductal transport-stage equine embryos. Biol Reprod. (1991) 45:540–3. doi: 10.1095/biolreprod45.4.540, PMID: 1751627

[42] Budik S Walter I Leitner MC Ertl R Aurich C. Expression of enzymes associated with prostaglandin synthesis in equine conceptuses. Animals (Basel). (2021) 11:1180. doi: 10.3390/ani11041180 33924239 PMC8074782

[43] Wang H Dey SK. Lipid signaling in embryo implantation. Prostaglandins Other Lipid Mediat. (2005) 77:84–102. doi: 10.1016/j.prostaglandins.2004.09.013 16099394

[44] Sharp DC. The early fetal life of the equine conceptus. Anim Reprod Sci. (2000) 60-61:679–89. doi: 10.1016/S0378-4320(00)00138-X, PMID: 10844234

[45] Dennis EA. Lipidomics joins the omics evolution. Proc Natl Acad Sci. (2009) 106:2089–90. doi: 10.1073/pnas.0812636106, PMID: 19211786 PMC2650110

[46] Wenk MR. The emerging field of lipidomics. Nat Rev Drug Discov. (2005) 4:594–610. doi: 10.1038/nrd1776 16052242

[47] Fahy E Subramaniam S Murphy RC Nishijima M Raetz CRH Shimizu T. Update of the LIPID MAPS comprehensive classification system for lipids. J Lipid Res. (2009) 50:S9–S14. doi: 10.1194/jlr.R800095-JLR200, PMID: 19098281 PMC2674711

[48] Xu T Hu C Xuan Q Xu G. Recent advances in analytical strategies for mass spectrometry-based lipidomics. Anal Chim Acta. (2020) 1137:156–69. doi: 10.1016/j.aca.2020.09.060, PMID: 33153599 PMC7525665

[49] Evans HC Dinh TTN Hardcastle ML Gilmore AA Ugur MR Hitit M. Advancing semen evaluation using Lipidomics. Frontiers in veterinary. Science. (2021) 8:601794. doi: 10.3389/fvets.2021.601794 PMC8085260 33937366

[50] Castro-Perez JM Kamphorst J Degroot J Lafeber F Goshawk J YU K. Comprehensive LC-MS E lipidomic analysis using a shotgun approach and its application to biomarker detection and identification in osteoarthritis patients. J Proteome Res. (2010) 9:2377–89. doi: 10.1021/pr901094j, PMID: 20355720

[51] Phan K He Y Pickford R Bhatia S Katzeff JS Hodges JR. Uncovering pathophysiological changes in frontotemporal dementia using serum lipids. Sci Rep. (2020) 10:3640. doi: 10.1038/s41598-020-60457-w, PMID: 32107421 PMC7046653

[52] Ye Q Cai S Wang S Zeng X Ye C Chen M. Maternal short and medium chain fatty acids supply during early pregnancy improves embryo survival through enhancing progesterone synthesis in rats. J Nutr Biochem. (2019) 69:98–107. doi: 10.1016/j.jnutbio.2019.03.015, PMID: 31063920

[53] Suematsu R Miyamoto T Saijo S Yamasaki S Tada Y Yoshida H. Identification of lipophilic ligands of Siglec5 and-14 that modulate innate immune responses. J Biol Chem. (2019) 294:16776–88. doi: 10.1074/jbc.RA119.009835, PMID: 31551352 PMC6851322

[54] Lim PS Sutton CR Rao S. Protein kinase C in the immune system: from signalling to chromatin regulation. Immunology. (2015) 146:508–22. doi: 10.1111/imm.12510, PMID: 26194700 PMC4693901

[55] Yang QE Ozawa M Zhang K Johnson SE Ealy AD. The requirement for protein kinase C delta (PRKCD) during preimplantation bovine embryo development. Reprod Fertil Dev. (2016) 28:482–90. 25116760 10.1071/RD14160

[56] Gastal MO Gastal EL Torres CA Ginther OJ. Effect of PGE2 on uterine contractility and tone in mares. Theriogenology. (1998) 50:989–99., PMID: 10734418 10.1016/s0093-691x(98)00202-7

[57] Baardman ME Kerstjens-Frederikse WS Berger RMF Bakker MK Hofstra RMW Plösch T. The role of maternal-fetal cholesterol transport in early fetal life: current Insights1. Biol Reprod. (2013) 88:24. doi: 10.1095/biolreprod.112.102442, PMID: 23153566

[58] Paré JF Malenfant D Courtemanche C Jacob-Wagner M Roy S Allard D. The fetoprotein transcription factor (FTF) gene is essential to embryogenesis and cholesterol homeostasis and is regulated by a DR4 element. J Biol Chem. (2004) 279:21206–16. doi: 10.1074/jbc.M401523200 15014077

[59] Roy R Belanger A. Elevated levels of endogenous pregnenolone fatty acid esters in follicular fluid high density lipoproteins support progesterone synthesis in porcine granulosa cells. Endocrinology. (1992) 131:1390–6. doi: 10.1210/endo.131.3.1505469, PMID: 1505469

[60] Raeside J Christie H Renaud R Waelchli R Betteridge K. Estrogen metabolism in the equine conceptus and endometrium during early pregnancy in relation to estrogen concentrations in yolk-sac fluid. Biol Reprod. (2004) 71:1120–7. doi: 10.1095/biolreprod.104.028712 15163615

[61] Choi SJ Anderson GB Roser JF. Production of free estrogens and estrogen conjugates by the preimplantation equine embryo. Theriogenology. (1997) 47:457–66. doi: 10.1016/S0093-691X(97)00004-6, PMID: 16727998

[62] Zavy MT Vernon MW Sharp DC Bazer FW. Endocrine aspects of early pregnancy in pony mares: a comparison of uterine luminal and peripheral plasma levels of steroids during the estrous cycle and early pregnancy. Endocrinology. (1984) 115:214–9. doi: 10.1210/endo-115-1-214, PMID: 6734514

[63] Quehenberger O Armando AM Brown AH Milne SB Myers DS Merrill AH. Lipidomics reveals a remarkable diversity of lipids in human plasma 1. J Lipid Res. (2010) 51:3299–305. doi: 10.1194/jlr.M009449, PMID: 20671299 PMC2952570

[64] Klein C Scoggin KE Ealy AD Troedsson MH. Transcriptional profiling of equine endometrium during the time of maternal recognition of pregnancy. Biol Reprod. (2010) 83:102–13. doi: 10.1095/biolreprod.109.081612, PMID: 20335638

[65] Klohonatz KM Coleman SJ Islas-Trejo AD Medrano JF Hess AM Kalbfleisch T. Coding RNA sequencing of equine endometrium during maternal recognition of pregnancy. Genes (Basel). (2019) 10:749. doi: 10.3390/genes10100749 31557877 PMC6826732

[66] Klohonatz KM Hess AM Hansen TR Squires EL Bouma GJ Bruemmer JE. Equine endometrial gene expression changes during and after maternal recognition of pregnancy1. J Anim Sci. (2015) 93:3364–76. doi: 10.2527/jas.2014-8826, PMID: 26440005

[67] Merkl M Ulbrich SE Otzdorff C Herbach N Wanke R Wolf E. Microarray analysis of equine endometrium at days 8 and 12 of pregnancy. Biol Reprod. (2010) 83:874–86. doi: 10.1095/biolreprod.110.085233, PMID: 20631402

[68] Smits K Gansemans Y Tilleman L Van Nieuwerburgh F Van De Velde M Gerits I. Maternal recognition of pregnancy in the horse: are MicroRNAs the secret messengers? Int J Mol Sci. (2020) 21:419. doi: 10.3390/ijms21020419, PMID: 31936511 PMC7014256

[69] Rudolf Vegas A Podico G Canisso IF Bollwein H Almiñana C Bauerssachs S. Spatiotemporal endometrial transcriptome analysis revealed the luminal epithelium as key player during initial maternal recognition of pregnancy in the mare. Sci Rep. (2021) 11:22293. doi: 10.1038/s41598-021-01785-3 34785745 PMC8595723

[70] Ribeiro E Greco L Bisinotto R Lima F Thatcher W Santos J. Biology of preimplantation conceptus at the onset of elongation in dairy cows. Biol Reprod. (2016) 94:97. doi: 10.1095/biolreprod.115.134908 26935601

[71] Seo H Choi Y Shim J Yoo I Ka H. Prostaglandin transporters ABCC4 and SLCO2A1 in the uterine endometrium and conceptus during pregnancy in pigs. Biol Reprod. (2014) 90:1–10. doi: 10.1095/biolreprod.113.114934 24695625

[72] Zeng S Bick J Kradolfer D Knubben J Flöter VL Bauersachs S. Differential transcriptome dynamics during the onset of conceptus elongation and between female and male porcine embryos. BMC Genomics. (2019) 20:679. doi: 10.1186/s12864-019-6044-z, PMID: 31462226 PMC6714402

[73] Ribeiro ES Santos JEP Thatcher WW. Role of lipids on elongation of the preimplantation conceptus in ruminants. Reproduction. (2016) 152:R115–26. doi: 10.1530/REP-16-0104, PMID: 27335133

[74] Sharma K Shi Y. The yins and yangs of ceramide. Cell Res. (1999) 9:1–10. doi: 10.1038/sj.cr.7290001, PMID: 10321684

[75] Moussatche P Lyons TJ. Non-genomic progesterone signalling and its non-canonical receptor. Biochem Soc Trans. (2012) 40:200–4. doi: 10.1042/BST20110638, PMID: 22260690

[76] Ménézo Y Lichtblau I Elder K. New insights into human pre-implantation metabolism in vivo and in vitro. J Assist Reprod Genet. (2013) 30:293–303. doi: 10.1007/s10815-013-9953-9, PMID: 23430228 PMC3607680

[77] Pawlak P Malyszka N Szczerbal I Kolodziejski P. Fatty acid induced lipolysis influences embryo development, gene expression and lipid droplet formation in the porcine cumulus cells†. Biol Reprod. (2020) 103:36–48. doi: 10.1093/biolre/ioaa045, PMID: 32318713 PMC7313259

[78] Chronopoulou E Harper JC. IVF culture media: past, present and future. Hum Reprod Update. (2015) 21:39–55. doi: 10.1093/humupd/dmu040 25035437

[79] Carnevale EM. The mare model for follicular maturation and reproductive aging in the woman. Theriogenology. (2008) 69:23–30. doi: 10.1016/j.theriogenology.2007.09.011, PMID: 17976712

[80] Lawson JM Salem SE Miller D Kahler A Van Den Boer WJ Shilton CA. Naturally occurring horse model of miscarriage reveals temporal relationship between chromosomal aberration type and point of lethality. Proc Natl Acad Sci USA. (2024) 121:e2405636121. doi: 10.1073/pnas.2405636121, PMID: 39102548 PMC11331123

[81] Zander-Fox D Villarosa L Mcpherson NO. Albumin used in human IVF contain different levels of lipids and modify embryo and fetal growth in a mouse model. J Assist Reprod Genet. (2021) 38:2371–81. doi: 10.1007/s10815-021-02255-5, PMID: 34114110 PMC8490560

[82] Desmet KLJ Van Hoeck V Gagné D Fournier E Thakur A O’Doherty AM. Exposure of bovine oocytes and embryos to elevated non-esterified fatty acid concentrations: integration of epigenetic and transcriptomic signatures in resultant blastocysts. BMC Genomics. (2016) 17:1004. doi: 10.1186/s12864-016-3366-y, PMID: 27931182 PMC5146907

[83] Almiñana C Bauersachs S. Extracellular vesicles: multi-signal messengers in the gametes/embryo-oviduct cross-talk. Theriogenology. (2020) 150:59–69. doi: 10.1016/j.theriogenology.2020.01.077 32088033

[84] Saadeldin IM Kim SJ Choi YB Lee BC. Improvement of cloned embryos development by co-culturing with parthenotes: a possible role of exosomes/microvesicles for embryos paracrine communication. Cell Reprogram. (2014) 16:223–34. doi: 10.1089/cell.2014.0003, PMID: 24773308 PMC4030698

[85] Rosenbluth EM Shelton DN Wells LM Sparks AET Van Voorhis BJ. Human embryos secrete microRNAs into culture media—a potential biomarker for implantation. Fertil Steril. (2014) 101:1493–500. doi: 10.1016/j.fertnstert.2014.01.058, PMID: 24786747

[86] Konstantynowicz-Nowicka K Harasim E Baranowski M Chabowski A. New evidence for the role of ceramide in the development of hepatic insulin resistance. PLoS One. (2015) 10:e0116858. doi: 10.1371/journal.pone.0116858, PMID: 25635851 PMC4312035

[87] Schaffer JE. Lipotoxicity: when tissues overeat. Curr Opin Lipidol. (2003) 14:281–7. doi: 10.1097/00041433-200306000-00008, PMID: 12840659

[88] Watt MJ Barnett AC Bruce CR Schenk S Horowitz JF Hoy AJ. Regulation of plasma ceramide levels with fatty acid oversupply: evidence that the liver detects and secretes de novo synthesised ceramide. Diabetologia. (2012) 55:2741–6. doi: 10.1007/s00125-012-2649-3, PMID: 22854889 PMC3576922

[89] Rego A Trindade D Chaves SR Manon S Costa V Sousa MJ. The yeast model system as a tool towards the understanding of apoptosis regulation by sphingolipids. FEMS Yeast Res. (2014) 14:160–78. doi: 10.1111/1567-1364.12096, PMID: 24103214

[90] Abbas HK Tanaka T Duke SO Porter JK Wray EM Hodges L. Fumonisin-and AAL-toxin-induced disruption of sphingolipid metabolism with accumulation of free sphingoid bases. Plant Physiol. (1994) 106:1085–93. doi: 10.1104/pp.106.3.1085, PMID: 12232389 PMC159634

[91] Crossman MW Hirschberg CB. Biosynthesis of phytosphingosine by the rat. J Biol Chem. (1977) 252:5815–9. doi: 10.1016/S0021-9258(17)40095-0 885884

[92] Madison KC Swartzendruber DC Wertz PW Downing DT. Sphingolipid metabolism in organotypic mouse keratinocyte cultures. J Invest Dermatol. (1990) 95:657–64. doi: 10.1111/1523-1747.ep12514333, PMID: 2123494

